# Seroprotection Against Hepatitis B Virus Among Healthcare Workers in a Tertiary Care Teaching Hospital in Uttarakhand

**DOI:** 10.7759/cureus.102106

**Published:** 2026-01-22

**Authors:** Malvika Singh, Prachi Gupta, Sulekha Nautiyal, Ranjana Rohilla, Rajender Singh

**Affiliations:** 1 Microbiology, Himalayan Institute of Medical Sciences, Swami Rama Himalayan University, Dehradun, IND; 2 Microbiology, Graphic Era Institute of Medical Sciences, Graphic Era University, Dehradun, IND; 3 Microbiology, Shri Guru Ram Rai Institute of Medical and Health Sciences, Shri Guru Ram Rai University, Dehradun, IND; 4 Microbiology, All India Institute of Medical Sciences, Rishikesh, Rishikesh, IND

**Keywords:** anti-hbs antibody titre, healthcare workers, hepatitis b vaccination, hepatitis b virus, occupational exposure, seroprotection

## Abstract

Introduction

Hepatitis B virus (HBV) infection is a significant occupational health hazard among healthcare workers (HCWs) due to frequent exposure to blood and body fluids. Although hepatitis B vaccination provides effective protection, post-vaccination antibody levels may decline over time, leading to inadequate seroprotection even in fully vaccinated individuals. Estimation of hepatitis B surface antibody (anti-HBsAb) titres serves as an indirect marker of immunity and helps identify hypo-responders and non-responders who remain at risk of infection.

Aims and objectives

The present study aimed to assess the seroprotection status against HBV among vaccinated HCWs in a tertiary care teaching hospital in Uttarakhand. The objectives were to determine the proportion of HCWs with protective anti-HBsAb titres and to evaluate the association of seroprotection with age, gender, and occupational category.

Methods

A cross-sectional study was conducted over 18 months (May 2023 to October 2024) at a tertiary care teaching hospital in Dehradun, Uttarakhand. A total of 2,132 fully vaccinated HCWs, including doctors, nurses, technicians, housekeeping staff, medical students, and other hospital personnel, were enrolled. Blood samples were collected, and serum anti-HBsAb titres were estimated using a commercially available enzyme-linked immunosorbent assay (ELISA). An anti-HBsAb titre ≥10 mIU/mL was considered protective. Statistical analysis was performed using Jamovi version 2.6.44. Descriptive statistics were used, and associations between seroprotection and demographic or occupational variables were analysed using the chi-square test, with p < 0.05 considered statistically significant.

Results

Among the 2,132 HCWs included in the study, 1,318 (61.8%) were females, and 814 (38.2%) were males, with a mean age of 35.77 years. Overall, 1,502 (70.5%) HCWs demonstrated protective anti-HBsAb titres (≥10 mIU/mL), while 630 (29.5%) had titres <10 mIU/mL. A statistically significant association was observed between hepatitis B seroprotection and age group (p = 0.002), occupation (p < 0.001), and gender, with females exhibiting significantly higher protective antibody titres than males (χ² = 6.86, p = 0.009). Seroprotection was highest among doctors (299, 80.81%), followed by technicians (195, 75.28%) and nurses (590, 74.77%). In contrast, housekeeping staff and medical students demonstrated comparatively lower levels of protective antibody response, with seroprotection observed in only 44 (31.65%) and 61 (40.66%), respectively.

Conclusion

A substantial proportion of fully vaccinated HCWs lacked adequate seroprotection against HBV. Routine assessment of anti-HBsAb titres, particularly during pre-employment screening, along with targeted booster vaccination strategies, is recommended to ensure optimal protection and reduce occupational risk of HBV infection in healthcare settings.

## Introduction

Hepatitis B virus (HBV) is a blood-borne pathogen of significant global public health importance. HBV belongs to the family Hepadnaviridae and has humans as its only known natural host. Transmission occurs primarily through exposure to infected blood and other body fluids, including semen, via routes such as unsafe medical practices, tattooing, and vertical transmission from mother to child during pregnancy or the perinatal period [1]. Following entry into the bloodstream, the virus targets hepatocytes and replicates within hepatic tissue [2]. HBV infection is associated with substantial morbidity and mortality, encompassing a wide clinical spectrum. The majority of infected individuals remain asymptomatic [3]. Acute HBV infection may present with hepatic inflammation and jaundice, whereas persistent infection can progress to chronic hepatitis B, leading to severe and potentially fatal complications such as liver cirrhosis and hepatocellular carcinoma [4].

Although non-immunised individuals in developing countries are particularly vulnerable to HBV infection, the virus represents a well-recognised occupational health hazard among non-immunised healthcare workers (HCWs). This increased risk is attributable to frequent occupational exposure to blood and other potentially infectious biological materials, placing HCWs at a considerably higher risk than the general population [5].

The prevalence of HBV infection is notably higher among HCWs involved in invasive clinical procedures, including gynaecologists, surgeons, dentists, and nurses, as well as personnel who routinely handle human specimens, such as laboratory technicians, compared with other categories of HCWs [6].

In recognition of this occupational risk, hepatitis B vaccination has been recommended for all HCWs by the Centers for Disease Control and Prevention (CDC) since 1997 [7]. The World Health Organization (WHO) recommends measurement of hepatitis B surface antibody (anti-HBs) levels six to eight weeks after completion of the three-dose vaccination schedule to assess immune response. An anti-HBs antibody concentration of ≥10 mIU/mL is considered protective. The presence of anti-HBs antibodies at an adequate titre indicates immunity against HBV infection [8].

Despite vaccination, anti-HBs antibody levels may gradually decline over time. Consequently, both partially vaccinated and fully vaccinated HCWs may develop sub-protective anti-HBs titres. According to the WHO, hepatitis B vaccination coverage among HCWs ranges from 67% to 79% in developed countries, compared with 18% to 39% in developing countries [9].

The WHO’s 2024 Global Hepatitis Report highlights an alarming increase in hepatitis-related mortality worldwide. Viral hepatitis has emerged as the second leading infectious cause of death globally, accounting for approximately 1.3 million deaths annually. Data from 187 countries indicate that hepatitis-related deaths rose from 1.1 million in 2019 to 1.3 million in 2022, with hepatitis B responsible for 83% of these deaths and hepatitis C accounting for the remaining 17% [10].

In light of the persistent burden of hepatitis B infection and the potential decline in vaccine-induced immunity, the present study was undertaken to assess the rate of seroprotection among vaccinated HCWs at our institution.

## Materials and methods

Study design

The present cross-sectional study was conducted at Shri Guru Ram Rai Institute of Medical and Health Sciences, Shri Mahant Indiresh Hospital, Dehradun, Uttarakhand, over a period of 18 months, from May 2023 to October 2024.

Ethical approval

Ethical approval for the study was obtained from the Institutional Ethics Committee of Shri Guru Ram Rai Institute of Medical and Health Sciences (Reference No.: SGRR/IEC/11/22). Written informed consent was obtained from all participants prior to enrolment in the study.

Inclusion criteria

All HCWs, including doctors, nursing staff, laboratory technicians, housekeeping staff, medical students, and other hospital personnel (pharmacists, clerks, ward attendants, and laboratory attendants), who had completed the full three-dose schedule of hepatitis B vaccination were included in the study. HCWs who were not fully vaccinated at the time of enrolment were included only after completion of the vaccination schedule.

Exclusion criteria

HCWs who did not provide informed consent, those who had not completed the hepatitis B vaccination schedule, and individuals with incomplete demographic or laboratory data were excluded from the study.

Sample collection

Blood samples were obtained from all enrolled participants two months after completion of the hepatitis B vaccination schedule, using sterile plain vacutainer tubes under strict aseptic conditions. The samples were centrifuged, and the separated serum was used for the detection of hepatitis B surface antibody (anti-HBsAb) titres in the serology section of the Department of Microbiology.

Data collection

Anti-HBsAb titres were measured using the EUROIMMUN Medizinische Labordiagnostika AG (Lübeck, Germany) enzyme-linked immunosorbent assay (ELISA) kit. The assay is based on the principle that microtitre plates pre-coated with highly purified hepatitis B surface antigen (HBsAg) capture anti-HBs antibodies present in the serum during the first incubation. After washing, the bound antibodies are detected using horseradish peroxidase (HRP)-labelled HBsAg, which binds to the second antigen-binding site of the captured antibodies. Following the substrate-chromogen reaction, the resulting optical density is measured using an ELISA reader and is directly proportional to the concentration of anti-HBs antibodies in the sample.

Data analysis

Statistical analysis was performed using Jamovi software, version 2.6.44. Descriptive statistics were used to summarise the data, with categorical variables expressed as frequencies and percentages (N (%)) and continuous variables presented as mean ± standard deviation (SD). Comparative analysis was conducted using the chi-square test to assess associations between hepatitis B seroprotection status and variables such as gender, professional designation, and age categories. A p-value of <0.05 was considered statistically significant.

## Results

A total of 2,132 fully vaccinated HCWs were included in the study. Among them, nurses constituted the largest professional group (789, 37.0%), followed by doctors (370, 17.35%) and technicians (259, 12.14%). The remaining participants comprised housekeeping staff, medical students, and other hospital personnel.

Overall, female HCWs participated in greater numbers than males. Among nurses, a marked female predominance was observed, with female nurses accounting for 647 (82.0%), while males constituted 142 (18.0%). In contrast, the technician category demonstrated male predominance, with male technicians comprising 175 (67.4%) and females comprising 84 (32.6%).

Gender-wise analysis within individual professional categories revealed statistically significant differences in most groups. A chi-square goodness-of-fit test demonstrated a significant deviation from an equal male-female distribution among nurses, technicians, other staff, housekeeping staff, and medical students (p < 0.001 for all). In contrast, no statistically significant gender difference was observed among doctors, with male doctors accounting for 198 (53.5%) and female doctors comprising 172 (46.5%) (χ² = 1.56, p = 0.21), indicating a relatively balanced gender distribution within this professional category (Table 1).

**Table 1:** Gender-wise distribution of the subjects tested for hepatitis B surface antibody titres (n = 2132). Figures in parentheses are %. Chi-square (χ²) goodness-of-fit test was applied. P < 0.05 was considered statistically significant. * Pharmacist, clerks, and ward and lab attendants.

Profession	Male, n (%)	Female, n (%)	χ²	p-value
Doctors	173 (46.8)	197 (53.2)	1.56	0.21
Nurses	143 (18.1)	646 (81.9)	319.7	<0.001
Technicians	174 (67.2)	85 (32.8)	31.8	<0.001
Others*	257 (60.5)	168 (39.5)	18.7	<0.001
Housekeeping	32 (23.0)	107 (77.0)	40.5	<0.001
Medical students	35 (23.3)	115 (76.7)	42.7	<0.001

The mean age of the study population was 35.77 years. The largest proportion of participants belonged to the 36-40 years age group (634, 30.0%), followed by those aged 31-35 years (592, 28.0%) and ≥40 years (449, 21.1%). The remaining participants were distributed across younger age groups. A chi-square test of independence demonstrated a statistically significant association between age group and hepatitis B seroprotection status (<10 IU/mL vs. ≥10 IU/mL), with higher seroprotection observed in the 36-40 years age group (p = 0.002) (Table 2).

**Table 2:** Age-wise distribution of hepatitis B surface antibody titres. Figures in parentheses are %. Chi-square (χ²) test of independence was applied. P < 0.05 was considered statistically significant. IU/ml: international units per millilitre.

Age (years)	<10 IU/ml	>=10 IU/ml	Total	χ²	p-value
20-25	75 (38.1%)	122 (61.9%)	197 (9.2%)	16.72	p = 0.002
26-30	84 (32.3%)	176 (67.7%)	260 (12%)		
31-35	157 (26.5%)	435 (73.4%)	592 (28%)		
36-40	166 (26.1%)	468 (74%)	634 (30%)		
>41	149 (33%)	300 (67%)	449 (21.1%)		

On observing anti-HBsAb titres, it was found that 95 (68.34%) and 89 (59.33%) of the housekeeping staff and medical students, respectively, showed <10 IU/ml anti-HBsAb titres. Whereas, the majority of the doctors, nurses, technicians, and other staff, including pharmacists, clerks, attendants, etc., had titres > 10 IU/ml.

It was observed that 299 (80%) of the doctors had >10 IU/ml of anti-HBsAb titres, followed by technicians and nurses at 195 (75.28%) and 590 (74.77%), respectively. There was a significant association between occupation and protective anti-HBsAb titre (p < 0.001) (Table 3).

**Table 3:** Distribution of hepatitis B surface antibody titres in the study population. Figures in parentheses are %. Chi-square (χ²) test of independence was applied. P < 0.05 was considered statistically significant. * Pharmacist, clerks, and ward and lab attendants. IU/ml: international units per millilitre.

Occupational categorization	>=10 IU/ml	<10 IU/ml	χ²	p-value
Doctors	299 (80.81%)	71 (19.18%)	195.59	p < 0.001
Nurses	590 (74.77%)	199 (25.22%)		
Technicians	195 (75.28%)	64 (24.71%)		
Others*	313 (73.64%)	112 (26.35%)		
Housekeeping	44 (31.65%)	95 (68.34%)		
Medical students	61 (40.66%)	89 (59.33%)		
Total	1502	630 (29.5%)		

As shown in Table 4, protective anti-HBs antibody titres (≥10 IU/mL) were observed more frequently among females (42.3%) compared to males (28.1%). Chi-square analysis demonstrated a statistically significant association between gender and hepatitis B antibody response (<10 IU/mL vs. ≥10 IU/mL) (χ² = 6.86, df = 1, p = 0.009).

**Table 4:** Comparison of hepatitis B surface antibody titres in reference to gender. Figures in parentheses are %. Chi-square (χ²) test of independence was applied. P < 0.05 was considered statistically significant. Anti-HBs: hepatitis B surface antibody; IU/ml: international units per millilitre.

Gender	Anti-HBs <10 IU/mL, n (%)	Anti-HBs ≥10 IU/mL, n (%)	Total	χ²	p-value
Female	416 (19.5)	902 (42.3)	1318	6.86	0.009
Male	214 (10.0)	600 (28.1)	814		
Total	630 (29.5)	1502 (70.5)	2132		

## Discussion

HCWs are at an increased occupational risk of acquiring blood-borne infections, including HIV, HBV, and hepatitis C virus (HCV), due to frequent exposure to blood and body fluids. Among these infections, hepatitis B is preventable through effective vaccination, underscoring the critical role of immunisation in mitigating occupational transmission and safeguarding HCWs [9].

In the present study, all enrolled participants had completed the full hepatitis B vaccination schedule. This contrasts with the findings of Sharma et al., who reported that 84% of participants were fully vaccinated while 16% were partially vaccinated [9]. Similarly, Batra et al. documented complete vaccination in only 49.6% of their study population, with an additional 4.3% being partially vaccinated [11]. These variations highlight differences in institutional vaccination policies, awareness levels, and access to occupational health services across healthcare settings.

A predominance of female participants was observed in the current study, accounting for 61.8% of the study population, with a female-to-male ratio of 1.6:1. This female predominance is consistent with the findings reported by Sharma et al. and Rao et al. [9,12]. In contrast, studies conducted by Mahawal et al. and Gembe et al. reported male predominance among vaccinated HCWs [13,14]. The mean age of participants in the present study was 35.77 years, with the largest proportion (30%) belonging to the 36-40-year age group, followed by 28% in the 31-35-year group and 21.1% aged 40 years and above. These findings differ slightly from those of Sharma et al., who reported a mean age of 30.26 years, with the predominant age group being 26-30 years, while a study conducted in Rwanda identified the predominant age group as 30-44 years [9,15].

In the present study, the highest proportion of participants with protective anti-HBs antibody titres (>10 IU/mL) was observed in the 36-40-year age group (74%). A statistically significant association was found between age group and anti-HBs antibody titre (p = 0.002). This association may, in part, reflect the higher representation of HCWs within this age group. In a similar study conducted at another tertiary care centre in Dehradun, the highest proportion of participants with protective titres (84.3%) belonged to the 31-35-year age group [9]. Conversely, a cross-sectional study from Tanzania reported that 91.4% of participants younger than 55 years had anti-HBs titres above the protective threshold, with a decline observed in individuals older than 55 years. The authors suggested consideration of booster doses in individuals above 55 years of age [14]. However, current evidence indicates that hepatitis B vaccines confer long-lasting, and often lifelong, protection in individuals who complete the full vaccination schedule, and routine booster doses are not generally recommended [16]. Exceptions may include specific high-risk populations, such as patients undergoing haemodialysis, who may require periodic monitoring and additional immunisation [17].

Occupational stratification revealed that protective anti-HBs antibody titres (>10 IU/mL) were most prevalent among doctors (80.81%), followed by technicians (75.28%) and nurses (74.77%). The higher antibody titres observed among doctors may be attributed to several interrelated factors. Repeated occupational exposure to HBV antigens, including subclinical exposure in high-risk clinical environments, may enhance secondary immune responses. Additionally, doctors are typically more knowledgeable about vaccination requirements, are vaccinated early during medical training, and are more likely to undergo regular occupational health evaluations, facilitating timely booster administration when indicated. Enhanced health literacy and greater engagement in preventive healthcare practices may further contribute to sustained immunological protection in this group [18]. The association between occupational category and anti-HBs antibody titre in the present study was highly significant (p < 0.001), consistent with findings reported by Sharma et al., who also observed higher seroprotection rates among doctors followed by nursing staff [9].

Despite complete vaccination, hypo-responsiveness, defined as anti-HBs antibody titres below 10 IU/mL, was observed in 29.5% of participants. Non-response to the hepatitis B vaccine has been documented in a subset of individuals, although the underlying mechanisms are multifactorial and not yet fully elucidated. Several host-related factors have been implicated in reduced vaccine responsiveness, including advanced age, male sex, obesity, smoking, excessive alcohol consumption, and chronic medical conditions such as diabetes mellitus, chronic kidney disease, liver disease, and HIV infection [19]. Genetic susceptibility also plays a significant role. Polymorphisms within the human leukocyte antigen (HLA) system, particularly HLA class II alleles, influence antigen presentation to CD4⁺ T-helper cells, a critical step in initiating an effective humoral immune response. Impaired antigen presentation or suboptimal T-helper cell activation may result in inadequate antibody production following vaccination. Studies have demonstrated higher rates of non-responsiveness among individuals homozygous for certain HLA alleles, including HLA-DRB1*0301, HLA-B8, DR3, DR7, and HLA-B44, suggesting a genetic basis for reduced seroconversion in susceptible individuals [20].

In the present study, female HCWs demonstrated significantly higher anti-HBs antibody titres compared to male HCWs (p = 0.009). This finding may be explained by inherent biological and hormonal differences influencing immune responses. Females generally exhibit stronger antibody-mediated immunity, partly due to the immunostimulatory effects of estrogen, which enhances B-cell activation and antibody production, while testosterone has been shown to exert immunosuppressive effects. Estrogen also promotes the secretion of cytokines such as interleukin-6 and interleukin-10, which play a role in augmenting vaccine-induced antibody responses [21]. Behavioural factors, including higher compliance with vaccination schedules and greater health-seeking behaviour among female HCWs, may further contribute to sustained protective antibody levels. However, these findings differ from those reported by Sharma et al., Baghianimoghadam et al., and Sahana et al., who found no significant association between gender and anti-HBs antibody titres [9,22,23].

The present study has certain limitations. It was conducted at a single centre and employed a retrospective design, which may limit the generalizability of the findings. Detailed information regarding potential confounding factors, such as obesity, smoking status, birth dose vaccination, alcohol consumption, and chronic medical conditions, including diabetes mellitus, chronic kidney disease, liver disease, and HIV infection, could not be comprehensively assessed. Future multicentric studies incorporating detailed risk factor analysis, along with HLA typing, are warranted to better elucidate determinants of vaccine responsiveness. Additionally, evaluation of revaccination strategies or booster dosing among non-responders may provide valuable insights into optimising long-term protection against hepatitis B infection among HCWs.

## Conclusions

HBV is a blood-borne virus. In healthcare settings, the major cause of accidental exposure to these viruses is incorrect handling of biomedical waste, with special reference to sharp waste. All newly joined HCWs must be given training on proper handling of biomedical wastes; also, the practice of continuous training on biomedical waste handling should be continued amongst the HCWs. All HCWs should be vaccinated and should be aware of their vaccinated status. The estimation of anti-HBs antibodies is an indirect measure of seroprotection against hepatitis B infection. It also gives insight into the prevalence of post-vaccination non-responders; such HCWs may be placed in areas where there is a low risk for acquiring hepatitis B infection. Therefore, hepatitis B immunisation should be compulsory for all HCWs. It is equally important to check the anti-HBs titre after six to eight weeks of vaccination. Also, the pre-employment assessment of anti-HBs antibodies titre of HCWs is highly recommended.
